# An intergenerational approach to parasitoid fitness determined using clutch size

**DOI:** 10.1038/s41598-022-09024-z

**Published:** 2022-03-25

**Authors:** Alena Samková, Jan Raška, Jiří Hadrava, Jiří Skuhrovec

**Affiliations:** 1 Department of Plant Protection, Faculty of Agrobiology, Food and Natural Resources, Czech University of Life Sciences Prague, Kamýcká 129, 165 00 Prague 6, Suchdol, Czech Republic; 2 Department of Zoology, Faculty of Science, Charles University, Viničná 7, 128 43 Prague 2, Czech Republic; 3 Institute of Entomology, Biological Centre, Czech Academy of Sciences, Branišovská 31, 370 05 České Budějovice, Czech Republic; 4 Crop Research Institute, Drnovská 507, 161 06 Praha 6, Ruzyně, Czech Republic

**Keywords:** Behavioural ecology, Population dynamics

## Abstract

Parasitoids, as important natural enemies, occur in high numbers and help maintain balance in natural ecosystems. Their fitness is traditionally studied as fertility based on the number of offspring in the F1 generation. Here, using gregarious parasitoids as models, we show that this traditional approach omits one important parameter: the clutch size–body size–fertility correlation among offspring. As a result of this correlation, when females adjust the number of offspring laid in a host, they determine not only the number of offspring produced but also the body size and reproductive potential of those offspring. Although parasitoid fertility has been determined several times from clutch size, here we use *Anaphes flavipes* to demonstrate the use of this relationship in an upgraded intergenerational approach to parasitoid fitness. We show that with a range of hosts simultaneously utilized by female parasitoids, identical fertility in the F1 generation can lead to distinctly different fertility values in the F2 generation. Even with the same number of hosts, lower fertility in the F1 generation can generate higher fertility in the F2 generation. Our approach provides an intergenerational perspective for determining individual fitness of gregarious parasitoids and new possibilities for the modelling of parasitoid population density.

## Introduction

The diversity of interspecific interactions between organisms is a remarkable natural phenomenon. One of these relationships is the parasitoid–host interaction, common in insects^1^. The females of parasitoids lay their eggs into various developmental stages of arthropods; their larvae develop in the host, feeding on its tissues and killing it before completing their own development^2^.

Parasitoids can be classed as solitary or gregarious^3^. The majority are solitary^4^ with females laying one or more eggs into a single host, but larval competition leads to only one surviving^5,6^. Gregarious development is derived and has evolved independently at least 43 times in 26 different families of Hymenoptera^7,8^. Gregariously developing larvae are more tolerant and/or less mobile than in solitary species^9,10^, thus allowing multiple individuals to mature from a single host^5,6^. Gregarious strategies can be based on multiple changes in larval phenotype, such as loss of aggression while retaining mobility retention of aggression with reduced mobility^11^. The tolerance model of Godfray^12^ assumes that when sharing the same host, tolerant gregarious larvae will always be killed by predatory solitary larvae (examined by Laing and Corrigan^13^). It is therefore assumed that to persist gregarious development must be associated with compensatory advantages.

One of the main benefits of the gregarious strategy is the efficient use of the host in the form of multiple individuals developing in a single host^14^. For this reason, however, the body size of the offspring is determined by “the mother's decision”, or how many offspring the mother allocates to each host^15,16^. Unlike predators, which consume several prey during larval development, parasitoids depend only on food resources obtained from a single host^2,17^. In optimal conditions, mothers should try to distribute their offspring among hosts so that each offspring reaches the same size^18^. However, with a scarcity of hosts, the mothers face a trade-off between the body size and number of their offspring: by laying a different clutch size (number of offspring per host) they choose either more, smaller offspring, or fewer, larger offspring^19^. The female not only chooses the number and size of her offspring, but also determines their fertility^20–22^. Indeed, in most gregarious parasitoids, the relationship between body size and fertility or fecundity is positive and linear^23,24^, especially in proovigenic species that mature as adults with a fixed number of eggs^25^.

Some clutch sizes or their combination are more advantageous than others^26^ and natural selection should select for behavioural responses that produce these advantageous combinations^26,27^. However, under different environmental conditions, females retain some plasticity in clutch size in relation to factors such as: (1) population density of parasitoids^28–30^ or including, in addition, the body size of females^31^ and (2) population density of host^23,32,33^ or host size^32^. It is clear that, for example, with fewer hosts, gregarious parasitoids may not reduce the number of offspring in the F1 generation due to increased clutch size, but at the cost of smaller offspring body size. In doing so, offspring with smaller body size from higher clutch sizes will give the mother lower potential fertility in the F2 generation^20^. These changes in the fertility of gregarious parasitoids are not visible from the perspective of the F1 generation, but only from the perspective of the F2 generation. However, the above^28,29^ and a large number of other studies^29,34–37^ discuss the impact of body size on offspring success in subsequent generations and contain data from which an intergenerational approach for parasitoid fitness could be determined: to the best of our knowledge, these studies examine clutch size variation only for the F1 generation.

Although body size-dependent fertility caused by differences in clutch size has been previously determined for several parasitoids^20,34^, in this study we determined the fertility of offspring from different clutch sizes for the parasitoid *A. flavipes* and demonstrated this relationship in an upgraded intergenerational approach to parasitoid fitness. Females of the gregarious idiobiont parasitoid *A. flavipes* lay 1–4 offspring per host (up to 7 offspring per host under extreme conditions), and in combination with sex ratio, a female has a choice of 35 combinations when a host egg is discovered, with certain combinations being more preferred^38^. Here, we have determined how fertility is obtained by maternal females in the F2 generation from offspring of real clutch size in the F1 generation assuming that the female has multiple hosts available at once. Host eggs were offered to wasps one at a time in a petri dish, however, each host egg was separated on approximately 1.5 cm of cereal leaf, thus wasps encountered host eggs sequentially. This situation probably does not correspond to reality because in nature hosts lay eggs in groups of two^39^ only in extreme cases of high host population density are eggs laid in clusters "in strings"^(pers. obs.)^. Here, however, we are concerned with the relationship between the distribution of wasp offspring among hosts and the fertility the wasp obtains from its offspring in the F2 generation. Using our intergenerational approach, we have shown that given the same number of parasitized hosts, the same fertility in the F1 generation can cause different fertility in the F2 generation, or even lower fertility in the F1 generation can cause higher fertility in the F2 generation. We discuss, based on data from our previous experiments, the contribution of an intergenerational approach to parasitoid fitness, whereby females with the same number of offspring in the F1 generation but with “*intentionally*” manipulation of clutch size based on differences in population density^40^ can obtain very different fertility in the F2 generation. Here, using Experiment 2, we then show that clutch size manipulation can also be “*unintentionally*” and females unintentionally manipulate the fertility of their offspring in the F2 generation.

## Results

### Exp. (1) determining fertility in the F2 generation

The fertility of the gregarious parasitoid *Anaphes flavipes* among different clutch sizes is shown in Table 1 (Suppl. Mat. 1).

**Table 1:** The fertility of the gregarious parasitoid *A. flavipes* among different clutch sizes (mean ± 95% confidence interval).

Clutch size	(a) Fertility of one offspring	(b) Fertility of all offspring per host	(c) Fertility of 12 offspring
1	19.3 ± 1.3	19.3	232 (100%)
2	13.8 ± 0.3	27.6	166 (72%)
3	11.2 ± 0.4	33.6	134 (58%)
4	10 ± 1.5	40	120 (52%)

The column "(c)" shows that the fertility of females in the F2 generation can be reduced by almost half, due to the different distributions of 12 offspring into clutch sizes.

**Figure 1:**
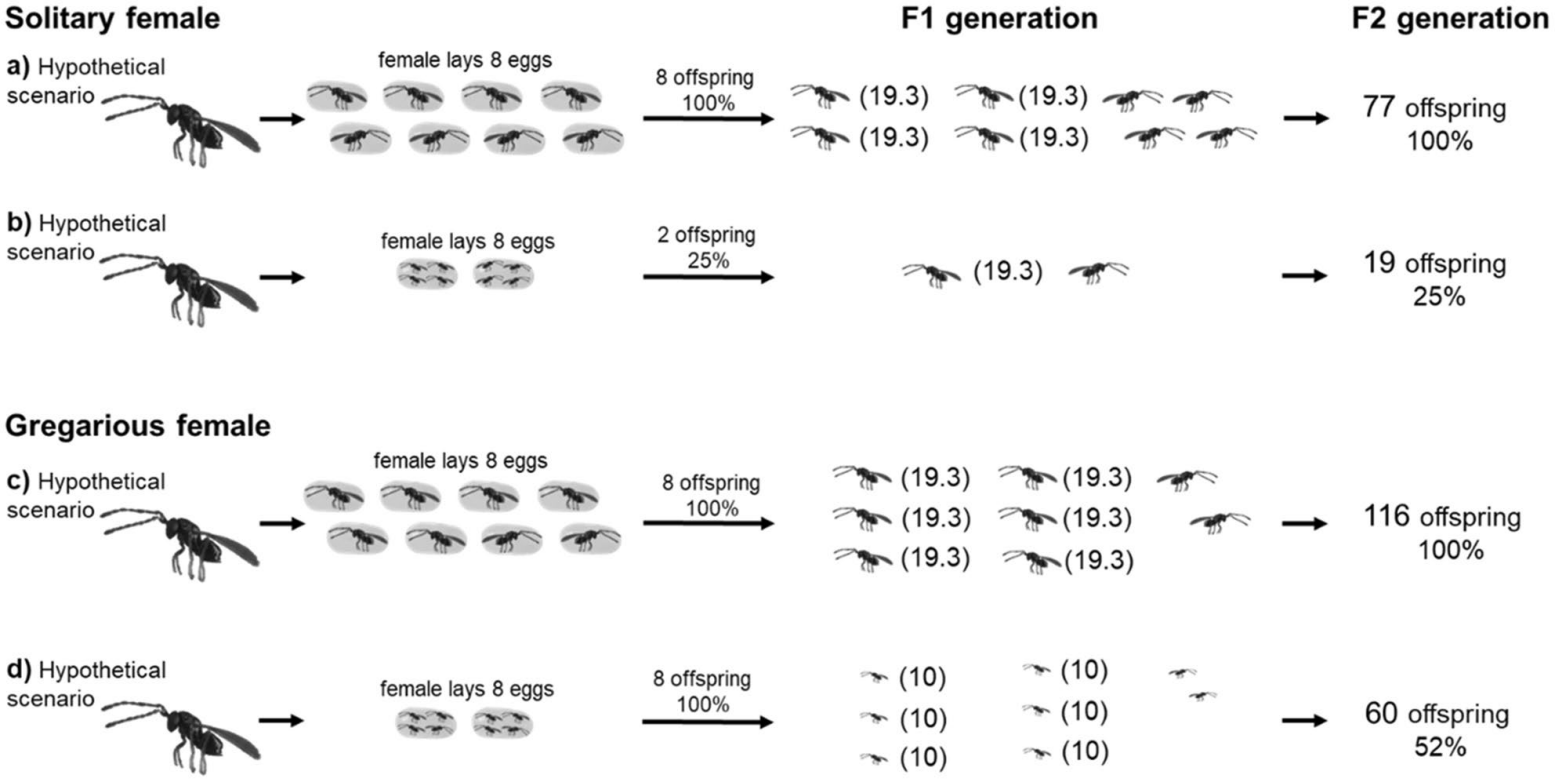
The different fertilities obtained in the F2 generation for solitary and gregarious parasitoids with different numbers of host eggs. *The fertility values shown in parentheses (explained in “Methods” section and Suppl. Mat. 3); The offspring sex ratio of solitary parasitoids is 1:1 (female:male; assuming random mating of individuals in the population^41^), and the offspring sex ratio of gregarious parasitoids is female biased 3:1 (female:male; reviewed for our model species *A. flavipes*^38^); females are shown as  (left oriented) and males as  (right oriented).

Using this, we determined the host utilisation by gregarious parasitoids compared to solitary parasitoids from the intergenerational approach (Fig. 1).

**Figure 2:**
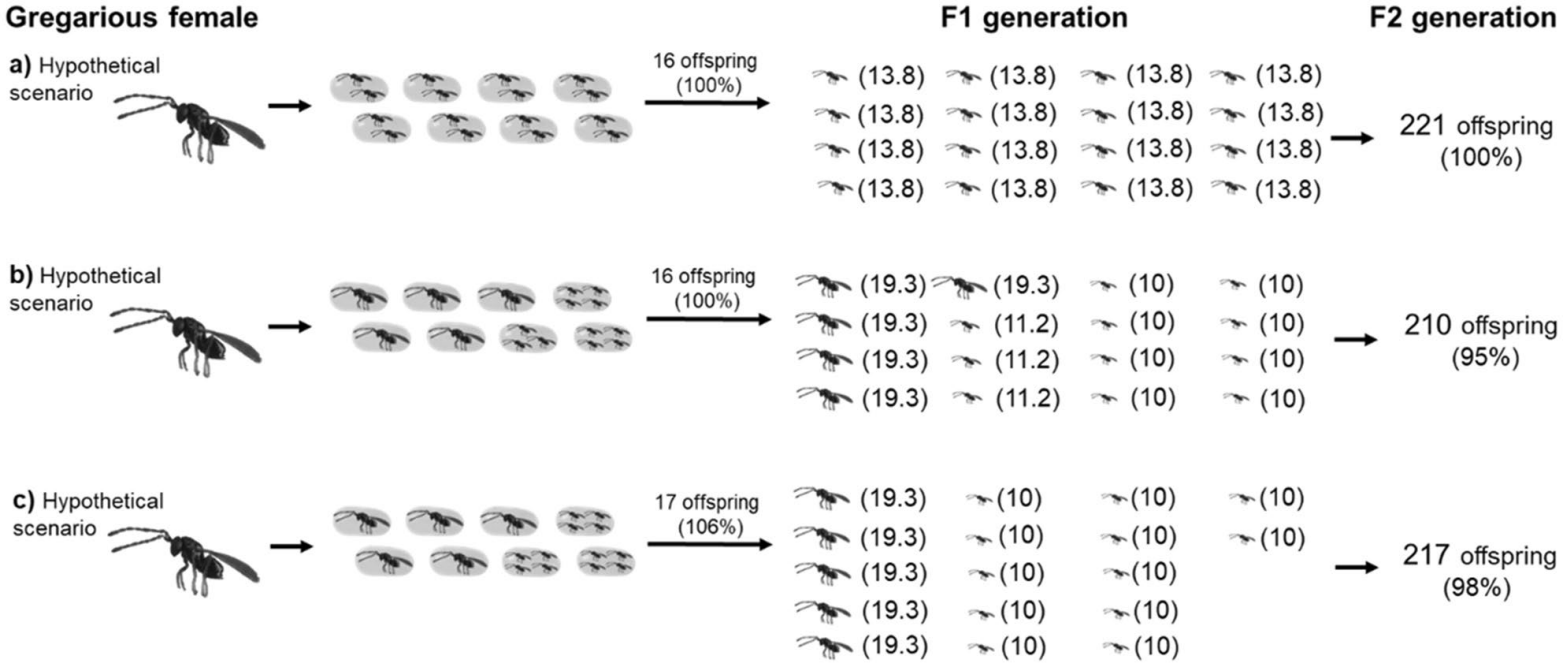
The different fertility values obtained in the F2 generation of females with the same number of host eggs and the same (**a**,**b**) or different fertility values in the F1 generation (**c**) (to simplify the model, the fertility values (shown in parentheses) are rounded (explained in “Methods” section); male offspring are not shown, explained in detail in the “Methods” section Exp. 2).

Using intergenerational approach, we have shown in Fig. 2 the advantage of certain clutch size combinations resulting in higher fertility in the F2 generation (Fig. 2).

We show, that the females can obtain high fertility values in the F2 generation from same fertility in the F1 generation (Fig. 2a,b) and that, even with the same number of hosts, lower fertility in the F1 generation can cause higher fertility in the F2 generation (Fig. 2c). Male offspring are not shown in Fig. 2 to make it clear that changes in fertility in the F2 generation may be due to the choice of favourable clutch size combinations and not to offspring sex ratio. The offspring sex ratio obviously affects fertility in the next generation^42^ where in general males should be as few as possible but able to fertilize all females among the offspring^43^.

### Exp. (2) effect of non-native hosts on clutch size

**Figure 3:**
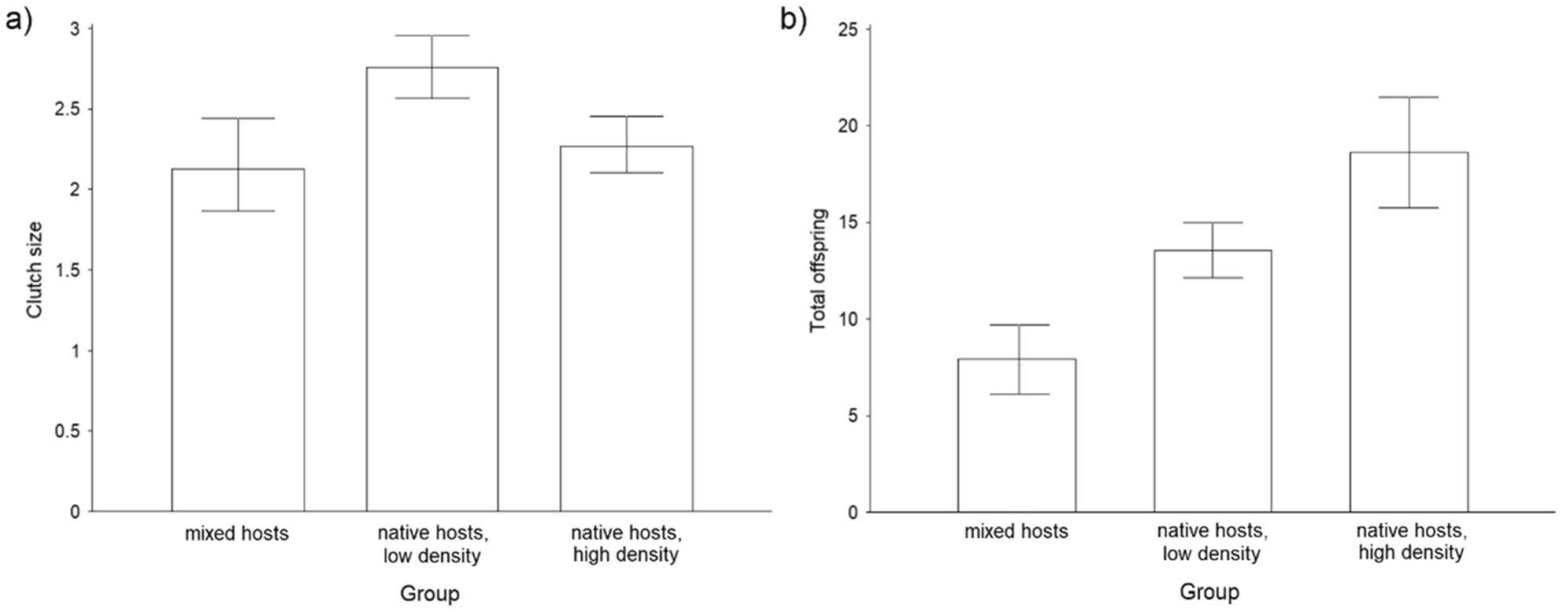
(**a**) Clutch size (i.e. offspring per parasitised host egg) and (**b**) total number of offspring per female *A. flavipes* under different conditions: mixed hosts (6 native host eggs and 6 non-native host eggs), native host, low host density (6 native host eggs); native host, high population density (12 non-native host eggs). Mean values and 95% confidence interval.

The clutch sizes of *A. flavipes* females (LMM, χ^2^ = 6.824, *p* = 0.009) with 12 host eggs available for parasitisation were lower and their fertility were higher (LM-sqrrt, F_(1,29)_ = 5.754, *p* = 0.023; Fig. 3) than those of females with 6 available host eggs due to the distribution of offspring among multiple hosts. Females with 6 native hosts and 6 non-native hosts in which the offspring were unable to complete larval development had lower clutch sizes than females with 6 native hosts (LMM, χ^2^ = 12.193, *p* < 0.001) and no significant difference in clutch size was observed compared to females with 12 native hosts (GLMM-p, χ^2^ = 0.516, *p* = 0.473; Fig. 3). The fertility in the group where females had 6 native and 6 non-native host for parasitisation were lower compared to both the group of females with 6 native hosts (LM, F_(1,24)_ = 20.356, *p* < 0.001) and that of females with 12 native host (LM, F_(1,33)_ = 32.583, *p* < 0.001; Fig. 3) (Suppl. Mat. 2).

## Discussion

The evolution of parasitoid clutch size is one of the most enduring topics in behavioural ecology and life history theory^8^. For gregarious parasitoids, some combinations of the clutch size confer more advantages than others and are therefore favoured by natural selection^27^.

Parasitoid body size should not only be considered in relation to fertility or longevity, as is most often the case^44^, but also, for example, in terms of effects on egg size^45^, foraging efficiency^21,35^ and competitiveness with other females^43^. In this study, we have clarified the advantage of clutch size combinations across and array of simultaneously presented hosts in the form of a higher fertility obtained in the F2 generation. Although a parasitoid’s fertility generally depends on the host’s population density and the number of eggs that a female has available^2^, we show that even with the same number of hosts and the same number of offspring, females can influence fertility in the F2 generation by choosing suitable clutch size combinations in the F1 generation (Fig. 2a,b). Moreover, with the same number of hosts, lower fertility in the F1 generation can cause a higher fertility in the F2 generation (Fig. 2b,c).

Based on our results, we propose the intergenerational approach, which takes into account the clutch size–body size–fertility correlation. Using this approach, we can obtain a more accurate view of the population densities of gregarious parasitoids compared to the traditional approach of maternal fertility^46,47^. For an illustration, we present a hypothetical scenario in which the same number of equally large *A. flavipes* parasitoids are released for biological control at two sites with different numbers of hosts (low and high). The population densities of the parasitoids, measured by abundance in the F1 generation (12–14 days after parasitoid release^38^), would be approximately the same at both sites, and differences would only be observed in the body size of the F1 offspring^40^. Using the intergenerational approach, we know that the size of the parasitoid population in the F2 generation (24–28 days after parasitoid release^38^) would be notably lower at the site with a low host population density due to the reduced body sizes of offspring in the F1 generation. Using knowledge of maternal clutch size decisions and characteristics of a particular site (low versus high host population density), one can predict future population denstity in these localities not only for the F1 generation, but also for the F2 generation (Fig. 1c,d).

We are aware that determining parasitoid fertility from clutch size requires extensive measurements that are difficult to obtain in the field. As an alternative, we propose obtaining knowledge of key factors, in the case of *A. flavipes* varying host population densities, intraguild predation^40^ or superparasitism^48^, whereby the parasitoid female “intentionally” changes the clutch size. Understanding these factors in a specific environment, we can expect a decrease or increase in clutch sizes and offspring fertilities, i.e. specifically predict their population density.

**Figure 4:**
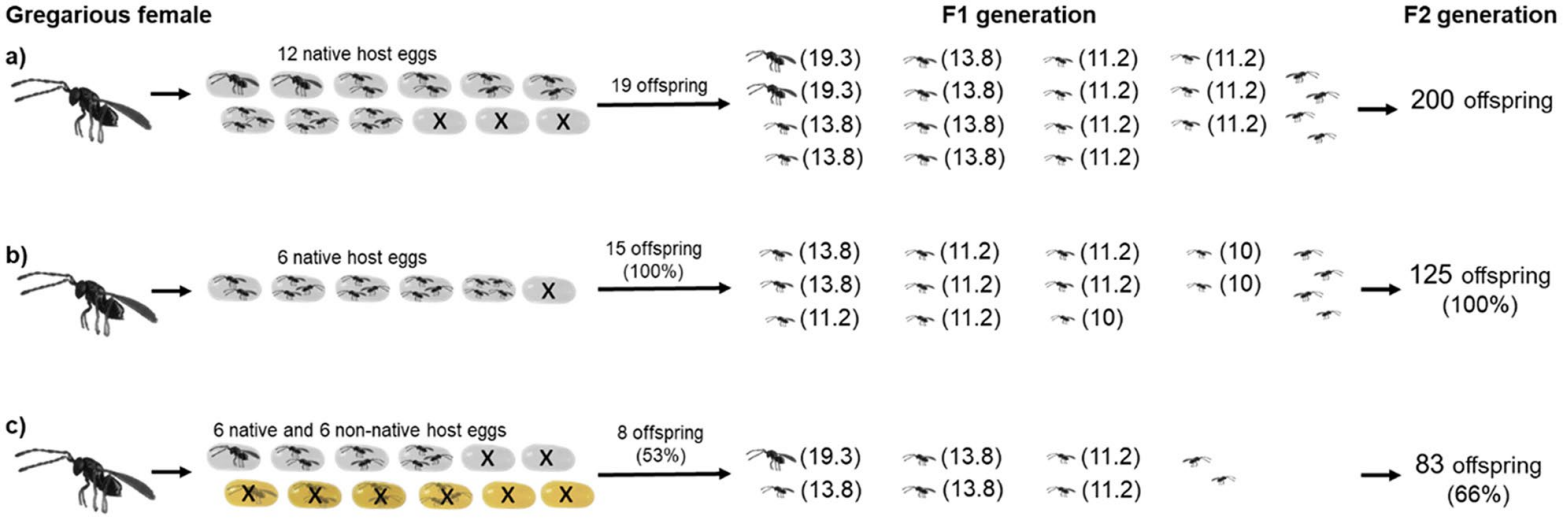
The different fertility values obtained for the F2 generations of three groups of females: (**a**) females with 12 available native host eggs; (**b**) females with 6 available native host eggs; and (**c**) females with 6 available native host eggs and 6 available non-native host eggs. *The fertility values shown in parentheses (explained in “Methods” section and Suppl. Mat. 1 and 3; the offspring sex ratio of *A. flavipes* is 3:1 (male:female)^38^; females are shown as  (left-facing) and males as  (right-facing).

Here, we have also found that clutch size can be affected “unintentionally” by the presence of non-native hosts in which the offspring of parasitoids *A. flavipes* are unable to complete larval development. Using the traditional one-generation approach, we would expect fertility to be reduced by almost half in locations with a non-native host (Fig. 4). However, from an intergenerational approach, we still expect reduced fertility on this locality, but only to a limited extent, because females, by non-random offspring distribution in both the non-native and native host, reduce their clutch size in the native host and thus increase the individual offspring fertility (Fig. 4c compared to Fig. 4b). If we apply the intergenerational approach, measuring certain clutch sizes, we find that in these circumstances the fertility of individuals in the F1 generation in the presence of a non-native host decreases by 53% (Fig. 4c), but among individuals in the F2 generation, fertility decreases by only 34% (Fig. 4c).

## Conclusion

The reproductive strategy of gregarious parasitoids is a interesting biological puzzle, as it represents a fertility-dependent, flexible life history strategy. This strategy brings benefits to its users in the form of the plasticity of clutch size combinations, by which the parasitoids can “intentionally” under pressure of external factors, such as host^23,34^ or parasitoids abundance^15,49^ host characteristics^32,45^, presence of predators^40^, and “unintentionally”^(this study)^ manipulate the fertility of offspring in the future generation. It is possible that by using an intergenerational approach to parasitoid fitness, it may be possible to better predict parasitoid population density affected by changes in clutch size. We suggest that determining fertility from clutch size is useful for important species of gregarious parasitoids, and propose the intergenerational approach as a model that may ensure more effective uses of parasitoids in biological plant protection or more successfully predict ecosystem stability.

## Materials and methods

### Parasitoids

*Anaphes flavipes* were reared from host eggs (*Oulema* spp.) collected in cereal fields in Prague (50.136° N, 14.363° E) from the end of April until the end of June in 2012, 2019 and 2020. The parasitised host eggs were stored in Petri dishes with moistened filter papers until adult wasps emerged. These “wild” wasps were used as an initial population from which the next generations of parasitoids were reared in an environmental chamber at 22 ± 2 °C with 40–60% relative humidity and under a 16:8-h (L:D) photoperiod. Subsequent generations of females and males were used for experiments. Mated females (not older than 24 h post-emergence) were placed in Petri dishes with host eggs. The females were not fed before the start of the experiment or during the experiment, and they had free access to water (modified from Samková et al.^19,40^).

### Host species

The non-native host species *Gastrophysa viridula* (DeGeer, 1775) and native hosts of the *Oulema* species complex were used in our experiment. *Oulema* species complex including two ecologically close species, *O. duftschmidi* (Redtenbacher, 1874) and *O. melanopus* (Linnaeus, 1758), which occur together in the same localities^50,51^ and which are only identifiable by genital preparation^52^. We^19,40^ and others authors^38,53^ consider it safe to use these two species in our study because we know from our previous experiments that their host eggs do not differ in length or weight^19^, *A. flavipes* wasps are not host-specific^51^, and their offspring from these two host species do not differ in size^19^ and do not show any morphological abnormalities^Samková et al. unpubl.^. In the current study, the host culture was established from adults collected in Prague (50.136° N, 14.363° E) and in Police n/Met (GPS: 50.527° N, 16.245° E). The adults were kept in plastic boxes with moistened filter papers, were fed, and had unlimited access to water. They were allowed to lay their eggs on leaves at 22 ± 2 °C, a relative humidity of 40–60% and a 16:8-h L:D cycle. We used host eggs no older than 24 h (modified from Samková et al.^19,40^).

### Laboratory experiments

All laboratory experiments were performed in Petri dishes (8.5 cm) in a thermal cabinet at 22 ± 2 °C and 40–60% relative humidity under a 16:8-h (L:D) photoperiod. In all experiments, each female was offered all host eggs to parasitize at once in a Petri dish, however, each host egg was offered to the wasp separately on about 1.5 cm of grain leaf, so the wasp met the host eggs one by one. Petri dishes with wasp and host eggs were kept for 8 h in the thermal cabinet, when self-superparasitation of host eggs was possible. Self-superparasitism has been observed in *A. flavipes* in our previous experiments and in the study Anderson and Paschke^38^. Individual parasitised host eggs were moved into 1.5-ml plastic tubes on the 9th or 10th day after parasitisation and stored at the same temperature in a thermal cabinet. The number and sex ratio of wasps that emerged from each parasitised host egg (referred to as clutch size in the text) were measured. The body size of the parasitoids was measured using temporary microslides according to established methodology^19^ (modified from Samková et al.^19,40^).

### Exp. (1) determining fertility in the F2 generation

Each female (n = 81) had 12 host eggs available for parasitisation for 8 h. We measured the body sizes of the females and their fertility values to determine the relationship between body size and fertility (the number of offspring developing in one host). After this, we measured the clutch size and body sizes of the offspring (n = 441) to determine fertility from the body size of *A. flavipes*. The part of the data from these experiments were used for studies (Samková et al.^19^) focused on the body size, fertility and factors influencing the clutch size of *A. flavipes* female parasitoids (Suppl. Mat. 1).

Using an intergenerational approach to *A. flavipes* fitness, we showed in hypothetical Fig. 2 the advantage of certain clutch size combinations in the form of differential fertility obtained in the F2 generation. In the hypothetical figure, the same number of hosts offered for parasitization to the maternal females is maintained, and the fertility in the F2 generation is shown only from the F1 generation females to show that the differential fertility obtained in the F2 generation is not affected by these factors.

### Exp. (2) effect of non-native hosts on clutch size

Each female had host eggs available for parasitisation for 8 h in three groups: (1) 12 native hosts (n = 19); (2) 6 native hosts (n = 11); and (3) 6 non-native and 6 native hosts (n = 15) (Suppl. Mat. 2).

### Simulation of an intergenerational reproductive approach

Fertility of offspring in all figures was measured using *A. flavipes*, see “Methods” section and Suppl. Mat. 1. Figures 2 and 3 are illustrative, depicting a hypothetical but possible scenario of offspring distribution in hosts. Figure 1 shows the offspring sex ratio of 1:1 (female:male) according to study Godfray^42^ for solitary parasitoids and 3:1 (female:male) according to Anderson and Paschke^38^ for gregarious parasitoids, specifically *A. flavipes*. The sex ratio of offspring is not shown in Fig. 2., because we want to emphasize that the differences in fertility in the F2 generation are due to maternal selection of advantageous clutch sizes in the F1 generation and not to differences in the sex ratio of offspring or by the distribution males into advantageous clutch sizes (e.g., one or two offspring developing in one host) will cause a reduction in the fertility of female offspring in disadvantageous combinations (e.g., 4 offspring developing in one host).

In Fig. 4, the distribution of offspring in host eggs (number and sex ratio) was determined using the frequencies of each clutch size in empirical data (Suppl. Mat. 3). The sex ratio of offspring did not differ significantly between groups, hence the 3:1 ratio used by Anderson & Paschke^38^ in the figures (Figs. 3 and 4).

### Statistical analysis

We estimated the effect of the F0 female reproductive strategy on F1-generation fertility in a two-step analysis. First, we carried out a linear regression of the effect of *A. flavipes* female wing length (body size) on fertility (both normally distributed) based on measurements of wasps used in a previous study^19^. The parameters of the regression line were calculated in Statistica 7 (StatSoft, Inc., 2004). In the second step, we calculated the hypothetical fertility of each female wasp used in the current study by applying the regression parameters to their known wing lengths. The total F1-generation fertility per host was calculated for all clutch sizes (1–4) by multiplying the clutch-size specific hypothetical fertility of a single female offspring by clutch-size specific mean number of female offspring per host. Since the ultimate aim of this model was to estimate the intergenerational fitness of the wasps, male fertility was considered to be zero.

Analyses testing the effects of the number of native/non-native hosts on reproduction of *A. flavipes* reproduction were performed in R 4.0.3^54^. For the number of offspring per dish, we used linear models (LM), the dependent variable was square-root transformed if necessary (labelled as "LM-sqrrt"). The clutch size was analysed by means of mixed effect linear models (LMM) or generalized mixed-effect linear models for Poisson distribution (GLMM-p), depending on distribution of analysed subset, with an ID specific for each dish as a random factor.

Mixed-effect models were built in R package lme4^55^ and analysed by their comparison with corresponding null models (including the random factor only) by means of likelihood ratio tests. The models were pairwise, including all three possible two-level subsets of the Group variable (levels: 6 native hosts; 12 native hosts; mixed hosts–6 native and 6 non-native ones), which was a single fixed factor in the models. 95% confidence intervals displayed in Fig. 3 were calculated in R 4.0.3^54^. 95% confidence intervals displayed in Fig. 3 were calculated in R 4.0.3^55^ for normal data distribution and R package DescTools for Poisson distribution (Group levels “12 native hosts” and “mixed hosts”).

## Supplementary Information

Supplementary Information 1.

Supplementary Information 2.

Supplementary Information 3.
